# From precision to strength: computer vision for suture quality assessment—an ex vivo pilot study

**DOI:** 10.1007/s00464-025-12441-6

**Published:** 2025-12-04

**Authors:** Roberto Spagnulo, Francesco Marzola, Federica Corso, Giovanni Distefano, Matteo Pescio, Kengo Hayashi, Federica Barontini, Giulio Dagnino, Kaspar Althoefer, Bruno Siciliano, Sebastien Ourselin, Alberto Arezzo

**Affiliations:** 1 Department of Surgical Sciences, University of Turin, Corso Dogliotti 14, 10128 Turin, Italy; 2 Department of Mechanical and Aerospace Engineering, Politecnico di Torino, Turin, Italy; 3 Department of Gastrointestinal Surgery, University of Kanazawa, Kanazawa, Japan; 4 Robotics and Mechatronics, University of Twente, Enschede, The Netherlands; 5 School of Engineering and Materials Science, Queen Mary University of London, London, UK; 6 Department of Electrical Engineering and Information Technology, University of Naples Federico II, Naples, Italy; 7 School of Biomedical Engineering and Imaging Sciences, King’s College London, London, UK

**Keywords:** Minimally invasive surgery, Quantitative suture assessment, Robotic-assisted surgery, Real-time intraoperative feedback, Surgical skill evaluation

## Abstract

**Background:**

Suturing is the cornerstone of surgical practice, yet its assessment continues to rely on subjective evaluation. As minimally invasive techniques become increasingly central in surgery, the demand for precision grows. In this context, the present pilot study aims to investigate whether spatial metrics of suture placement can quantitatively reflect mechanical performance and to determine how operator experience and surgical platform influence technical outcomes.

**Methods:**

Fifteen participants, stratified by prior experience in minimally invasive surgery, performed standardized suturing tasks on porcine rectal specimens using three platforms: conventional laparoscopy, the daVinci Research Kit (dVRK), and the Flex robotic endoscope. Quantitative Suture Assessment was conducted by extracting spatial features (e.g., distance variability between consecutive stitches), while mechanical resistance was evaluated via intraluminal burst pressure. Statistical analyses included correlation and regression modeling to assess the relationship between spatial metrics and burst pressure, as well as comparative analyses across platforms and experience levels.

**Results:**

Mean burst pressures measured 17.38 ± 6.54 mmHg for laparoscopy, 15.99 ± 7.69 mmHg for dVRK, and 13.60 ± 10.08 mmHg for Flex. Masters recorded a mean burst pressure of 17.95 ± 9.43 mmHg, while Advanced 13.89 ± 7.41 mmHg and Beginners 15.13 ± 7.64 mmHg. Laparoscopy achieved higher burst pressures than Flex, and dVRK outperformed Flex among Beginners. Laparoscopy and dVRK were faster than Flex, with Masters completing tasks more rapidly across all platforms.

Spacing irregularity was negatively correlated with burst pressure (p < 0.05). However, the XGBoost model trained on all variables exhibited poor performance (R^2^ = -0.42, MSE = 93.7), and high multicollinearity.

**Conclusions:**

Platform-specific scores emerged with limitations. While spatial metrics correlate with mechanical resistance, they appear insufficient as standalone indicators of suture quality. Ultimately, this approach could pave the way toward intelligent, intraoperative systems capable of delivering real-time feedback, with implications for both surgical education and quality assurance.

**Supplementary Information:**

The online version contains supplementary material available at 10.1007/s00464-025-12441-6.

Despite continuous innovations in surgical technology, suturing remains a fundamental determinant of clinical success across procedures. High-quality suturing ensures tissue approximation and promotes healing, thereby minimizing postoperative complications including infection [1], anastomotic leakage, and dehiscence [2]. While the global 30-day cumulative incidence of suture dehiscence is estimated at approximately 1,8% in gastrointestinal surgery operations [3], this figure rises dramatically in specific high-complexity contexts. For instance, in right hemicolectomy, a low anastomotic suture resistance is associated with leaks in over 7% of cases [4]. In Transanal Endoscopic Microsurgery (TEM), instead, dehiscence affects over 17% of patients [5] and up to 32% of those receiving neoadjuvant chemoradiotherapy [6]. These complications may lead to reoperations, infections, prolonged hospital stays, and up to a 30% increase in healthcare costs [7]. Thus, especially in high-risk scenarios, reducing the incidence of dehiscence becomes an urgent clinical need [8].

Although pivotal, the assessment of suture quality is still predominantly subjective. Evaluations are typically based on visual inspection and operator experience, lacking reproducibility and standardization [9, 10]. This subjectivity poses challenges in both clinical practice and surgical education [11]. On an epistemological level, universally accepted, objective criteria to define and measure the quality of a suture are currently undefined. Even structured performance studies [12, 13] have failed to demonstrate significant differences in outcomes between operators of varying expertise, highlighting the challenge in identifying traits of the excellent suture.

Minimally Invasive Surgery (MIS), now widely regarded as the gold standard in many general surgery procedures, poses several technical challenges, including diminished depth perception [14], constrained instrument articulation [15], and attenuated haptic feedback [16]. This further amplifies the demand for accurate and reliable suturing [17, 18], reinforcing the importance of reproducible, quantitative methods to evaluate technical performance [19, 20]. In response, Quantitative Suture Assessment (QSA) has emerged as a framework for measuring variables such as stitch spacing, consistency of alignment, and mechanical strength [21, 22], as well as surgical knot resistance [23], needle driver dynamics [24], and interaction forces with the tissue [25]. While promising in concept, existing QSA methodologies remain confined to research settings due to challenges in standardization, integration into clinical workflows, and relationship with surgical data.

Developing objective suture assessment tools is equally critical for surgical training [26]. Quantified metrics may inform skill progression, provide feedback within simulation environments [27], and support cross-platform comparisons of surgical performance. As robotic platforms and AI-enabled systems become more prevalent [28], integrating structured assessment protocols into both training and intraoperative workflows will be essential for enhancing procedural safety and effectiveness [29]. Importantly, assessment protocols should be system-independent, functioning without additional sensors or hardware that may impede clinical adoption. Standardized, video-based frameworks can enable broad implementation across surgical settings while preserving accuracy and reliability.

## Materials and methods

**Fig. 1:**
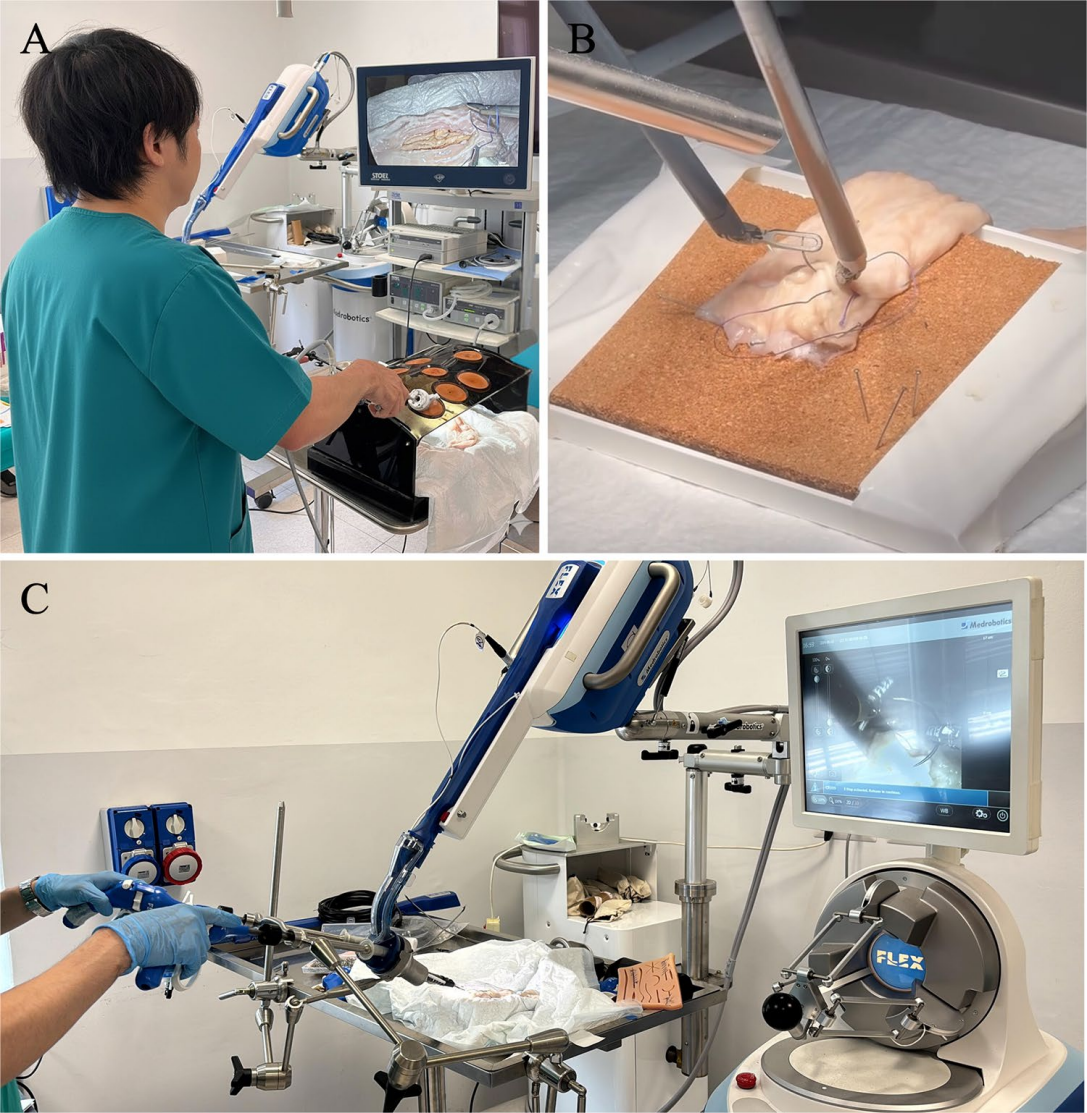
Minimally Invasive Surgical Platforms, Laparoscopy **A**. DaVinci Research Kit **B**. Flex Endoscope **C**

The present experimental design ensured standardization of all task conditions. Platforms included the da Vinci Research Kit (dVRK) by Intuitive Surgical (Sunnyvale, CA, USA), the Flex® System by Medrobotics (Raynham, MA, USA), and a custom laparoscopy simulator (Fig. 1).

Fifteen participants from the University of Turin, Department of Surgery were enrolled and stratified into three groups based on prior surgical experience: Masters (attending surgeons with over 100 minimally invasive procedures), Advanced (senior residents with more than 30 procedures), and Beginners (junior residents and senior medical students, with less than 30 procedures). Each participant completed one standardized suturing task per platform, yielding a total of 45 evaluated procedures. To control for potential order effects and skill-transfer bias, the sequence of platform exposure was systematically counterbalanced. Participants were randomly assigned to one of three order groups:

Group 1: Platform A → Platform B → Platform C.

Group 2: Platform B → Platform C → Platform A.

Group 3: Platform C → Platform A → Platform B.

This system ensured that each platform appeared equally often in the first, second, and third positions across the cohort. Participants were initially given a 10-min familiarization period to become acquainted with the surgical instruments, environment, and tissue manipulation techniques on each platform.

### Specimens and suturing protocol

Fresh, defrosted porcine sigmoid and rectal segments were utilized following local institutional standards for handling biological material (ASL Città della Salute e della Scienza di Torino – Presidio Molinette). Only bowel segments located within 22 cm from the anal verge were included to minimize tissue variability [30]. A standardized 3-cm horizontal incision was made with a scalpel in each sample. Tissues were hydrated by spraying water to maintain elasticity and prevent drying. A new tissue segment was used for each procedure to ensure consistency and repeatability.

Participants were instructed to perform a running suture using the “small bites” technique [31] (5 mm between stitches and 5 mm from the incision edge), consisting of 7 stitches followed by a closing knot. Coated Vicryl® (Ethicon Inc., Somerville, NJ, USA) 2–0 was used uniformly across all procedures. The thread length was adjusted according to platform-specific constraints, (i.e., instrument working range, operational workspace and ergonomic requirements): 20 cm for the dVRK, 22 cm for laparoscopy, and 24 cm for Flex.

### Evaluation metrics

Four ad hoc, objective metrics were designated and analyzed. The primary endpoint of this study was to evaluate the impact of operator experience level and surgical platform on suturing performance, as measured by spatial accuracy, procedural duration, and validated subjective feedback﻿. The secondary endpoint was to investigate the relationship between suture placement accuracy and burst pressure to assess whether specific spatial metrics were predictive of suture resistance to internal pressure.

### Procedural time

Procedural time was recorded using a digital chronograph, measured from the initial to the final movement of any robotic/laparoscopic arm.

### Suture accuracy

Suture accuracy was evaluated in each trial via two primary values: the distance between adjacent needle entry or exit points (point to point, PP) and the distance from each entry or exit point to the incision edge (point to margin, PM). Additional parameters were derived to assess suture consistency and alignment, for a total of eighteen distance metrics (Table 1).

**Table 1:** Mathematical Definition of PM- and PP-derived Distance Metrics

**Variable**^a^	**Definition**	**Formula**
PM max	Maximum horizontal point-to-midline distance	$\mathrm{max}\left(P{M}_{i}\right)$
PM max error	Maximum deviation from ideal horizontal spacing	$\mathrm{max}\left||P{M}_{i}|-5\text{ mm}\right|$
PM mean	Mean horizontal distance	$\frac{1}{n}\sum |P{M}_{i}|$
PM mean error	Mean deviation from ideal horizontal spacing	$\frac{1}{n}\sum \left||P{M}_{i}|-5\text{ mm}\right|$
PM std	Variability in horizontal distance	$\sqrt{\frac{1}{n}\sum {\left(|P{M}_{i}|-\overline{PM}\right)}^{2}}$
PM std error	Variability in deviation from horizontal ideal spacing	$\sqrt{\frac{1}{n}\sum {\left(\left||P{M}_{i}|-5\text{ mm}\right|-{\overline{PM}}_{\mathrm{error}}\right)}^{2}}$
PP max	Maximum vertical point-to-point distance	$\mathrm{max}\left(P{P}_{i}\right)$
PP max error	Maximum deviation from ideal vertical spacing	$\mathrm{max}\left||P{P}_{i}|-5\text{ mm}\right|$
PP mean	Mean vertical distance	$\frac{1}{n}\sum |P{P}_{i}|$
PP mean error	Mean deviation from ideal vertical spacing	$\frac{1}{n}\sum \left||P{P}_{i}|-5\text{ mm}\right|$
PP std	Variability in vertical distance	$\sqrt{\frac{1}{n}\sum {\left(|P{P}_{i}|-\overline{PP}\right)}^{2}}$
PP std error	Variability in deviation from vertical ideal spacing	$\sqrt{\frac{1}{n}\sum {\left(\left||P{P}_{i}|-5\text{ mm}\right|-{\overline{PP}}_{\mathrm{error}}\right)}^{2}}$
P all max	Maximum of all distances	$\mathrm{max}\left(P{M}_{i};P{P}_{i}\right)$
P all max error	Maximum deviation from all ideal spacing	$\mathrm{max}\left(\left||P{M}_{i}|-5\text{ mm}\right|;\left||P{P}_{i}|-5\text{ mm}\right|\right)$
P all mean	Mean of all distances	$\frac{1}{2n}\sum \left(|P{M}_{i}|+|P{P}_{i}|\right)$
P all mean error	Mean deviation from all ideal spacing	$\frac{1}{2}\left(\frac{1}{n}\sum \left||P{M}_{i}|-5\text{ mm}\right|+\frac{1}{n}\sum \left||P{P}_{i}|-5\text{ mm}\right|\right)$
P all std	Overall variability in distances	$\frac{1}{2n}\sum \left[{\left(|P{M}_{i}|-\overline{PM}\right)}^{2}+{\left(|P{P}_{i}|-\overline{PP}\right)}^{2}\right]$
P all std error	Variability in deviation from all ideal spacing	$\sqrt{\frac{1}{2n}\sum \left[{\left(\left|P{M}_{i}-5 {\mathrm{mm}}\right|-{\overline{PM}}_{\mathrm{error}}\right)}^{2}+{\left(\left|P{P}_{i}-5 {\mathrm{mm}}\right|-{\overline{PP}}_{\mathrm{error}}\right)}^{2}\right]}$

^a^Let PM_i_ denote the horizontal (point-to-midline) distance for stitch i, PP_i_ the vertical (point-to-point) distance between stitches i and i + 1, and n the number of measurements. “Max” represents the highest observed value; “mean” the arithmetic mean; “std” the standard deviation, “error” the difference between the observed value and the instructed gold standard (i.e., small bites of 5 mm each); “horizontal” stays for entry or exit point to incision; “vertical” for entry or exit point to the, respectively, consecutive entry or exit point; “all” indicates that both PM- and PP-based distances have been taken into account (e.g., PM_mean_error is the mean variation of horizontal, entry/exit point-to-incision distances from the 5-mm gold standard)

All distances were obtained from high-resolution images of the sutures, captured under standardized conditions using an Apple iPhone 15 Pro camera, with consistent lighting and fixed camera distance. To ensure reliable extraction of spatial metrics, a 3 × 3 checkerboard grid was incorporated into each image as a geometric reference. This allowed to establish a robust scale for translating pixel-based measurements into real-world distances.

**Fig. 2:**
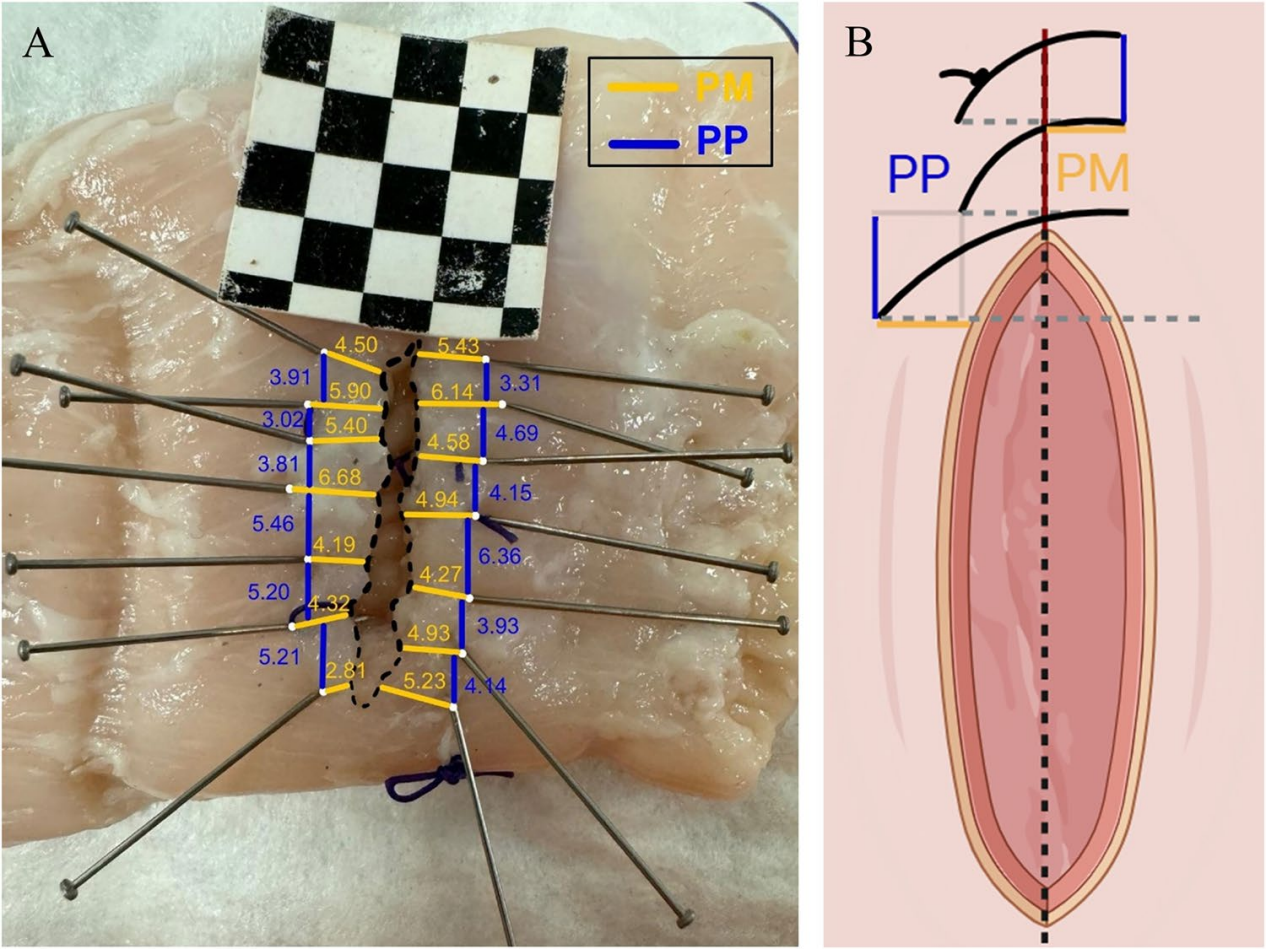
Post-processing Image and Sketch, Suture image after annotation and distance analysis **A**. Needle entry and exit points were marked in white; point-to-midline distances in yellow; point to consecutive point distances in blue. Conceptualized version **B**

Prior to image analysis, suture threads were cut and carefully removed to ensure uniform tissue apposition and coplanar alignment of the opposing flaps. To facilitate accurate digital processing, all needle entry and exit points were meticulously marked on the specimen using fine metal pins. These anatomical landmarks and corresponding incision edges were identified and marked across all sutures using MATLAB’s ImageLabeler toolbox (Fig. 2). Annotations were conducted collaboratively by a multidisciplinary team comprising experienced surgeons, biomedical engineers, and trained medical students. Following the initial labeling, a cross-validation step was conducted, in which team members independently reviewed each image.

### Burst pressure

**Fig. 3:**
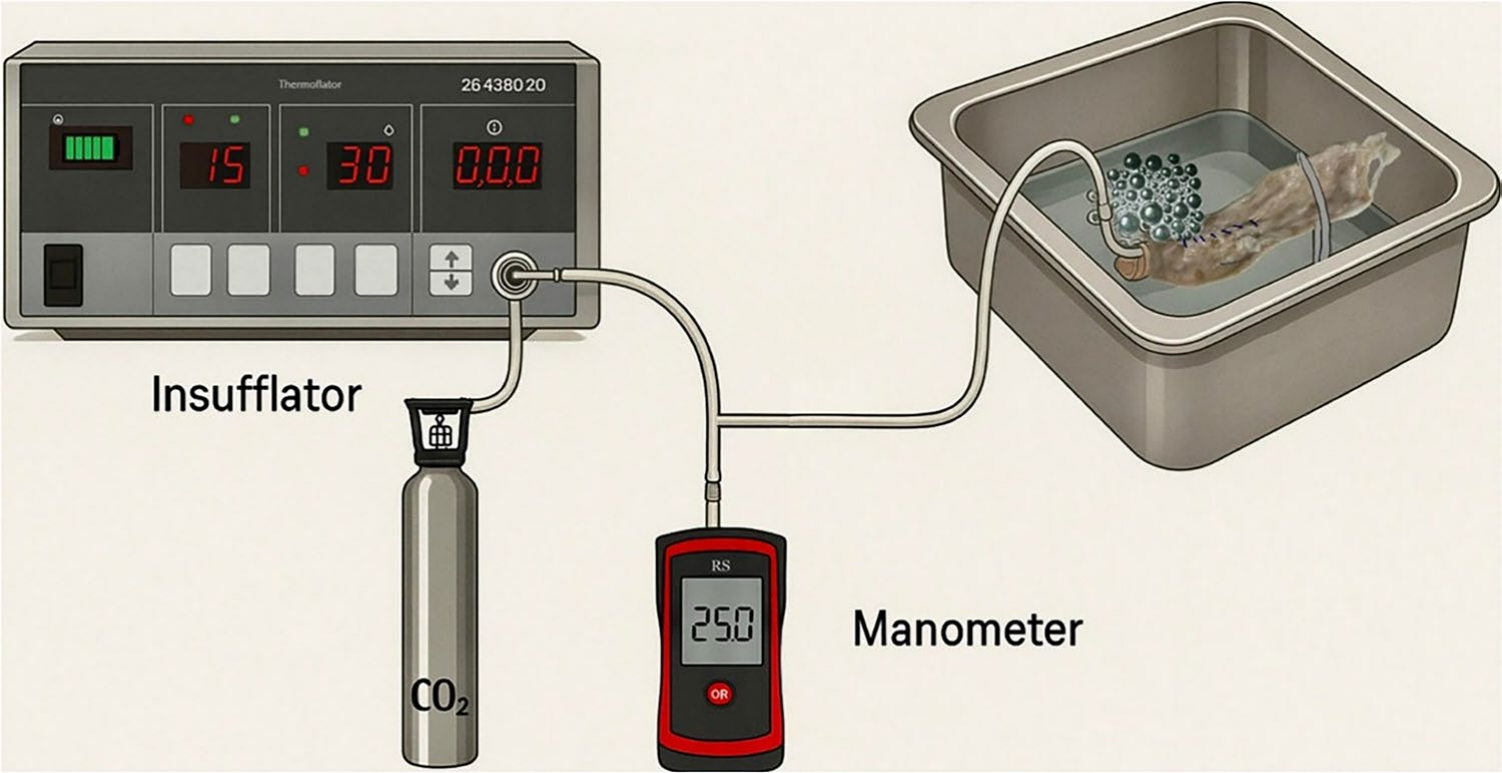
Experimental Setup In the illustrated pneumatic circuit, a carbon dioxide pump insufflates gas into the internal chamber created in the sutured rectum. A high-resolution manometer is connected in series to detect changes in pressure inside the circuit

Suture mechanical resistance was evaluated through burst pressure measurement [32–34]. While excessive tension in the suture thread may compromise tissue perfusion and healing due to ischemia, insufficient approximation can result in leakage and, thus, impaired recovery [35]. A closed pneumatic circuit enabled precise measurements via a high-resolution RS MH 5130 manometer (Corby, UK), a carbon dioxide (CO₂) pump—Karl Storz Endoscope 26,432,020 (Tuttlingen, Germany)—and an inflated chamber: the sutured rectum segment (Fig. 3). CO₂ insufflation was incrementally regulated, maintaining a pressure increase of less than 1 mmHg/s to avoid sudden stress on the tissue. One end of the bowel was sealed using a Kelly clamp, while the other end was connected to the pressurization system and closed with elastic bands to prevent leakage. The entire sutured rectum was submerged, and insufflation continued until the very first air bubbles appeared along the suture line, indicating initial loss of seal. Burst pressure was defined as the manometer reading at that precise moment. Measurements were captured by reviewing video recordings of both the specimen and the manometer display frame by frame.

### Subjective feedback

Following each procedure, participants completed two standardized, paper-based questionnaires to assess subjective workload and perceived usability. The NASA Task Load Index (NASA-TLX) [36] was employed to evaluate six workload descriptors, including mental demand, physical demand, temporal demand, performance, effort, and frustration. Each dimension was rated on a 10-point scale and subsequently categorized as low (1–3), moderate (4–6), or high (7–10) [37].

Perceived system usability was measured using the System Usability Scale (SUS) [38], a validated 10-item instrument rated on a 5-point Likert scale. Final SUS scores, ranging from 0 to 100, were interpreted as follows: < 50 indicated low usability, 50–69 moderate usability, 70–89 good usability, and 90–100 excellent usability. Scores were computed by subtracting 1 from the raw scores of odd-numbered items and subtracting even-numbered item scores from 5. The adjusted values were summed and multiplied by 2.5 to yield the final usability score [39].

#### Data analysis

All data were analyzed using Python. Descriptive statistics were computed for each metric. Intergroup comparisons (in terms of experience levels and platforms) were performed using appropriate statistical tests (i.e., t-tests, ANOVA), depending on the data distribution and sample homogeneity. A significance threshold of p < 0.05 was applied.

To evaluate how platform type and operator experience influence performance, analyses were conducted using suture burst pressure, task duration, and subjective feedback as outcome metrics. Since several variables exhibited non-normal distributions, Kruskal–Wallis tests were used to assess global differences, followed by Dunn’s post hoc comparisons. A retrospective power analysis was also conducted to evaluate sample adequacy. Subsequently, outliers were identified and removed using the interquartile range (IQR) method to minimize the influence of extreme values on group comparisons.

To assess whether suture spatial precision influences mechanical integrity, the relationship between burst pressure and 18 suture-derived spatial metrics was analyzed in two steps. Given the potential for inter-correlation among spatial features, we first assessed collinearity between features and their correlation with the target variable to perform systematic feature selection (Supplementary Table). This foundational approach enabled identification of the most discriminating metrics from a comprehensive set of parameters. To ensure the reliability of the observed correlations beyond their statistical significance, a power analysis was conducted.

Secondly, features exhibiting statistically significant correlations with burst pressure (p < 0.05) were selected and evaluated using a machine learning framework to determine their predictive power; a regression analysis was performed using eXtreme Gradient Boosting (XGBoost) combined with Repeated K-fold Cross-Validation (k = 5 splits, 10 repeats) to ensure robust and generalizable performance estimates. In each iteration, the model was trained on 80% of the data and validated on the remaining 20%, with different random partitions at every round. Model performance was evaluated using multiple metrics: Mean Squared Error (MSE), R-squared (R^2^), and Mean Absolute Error (MAE). SHAP (SHapley Additive exPlanations) analysis was then used to quantify the contribution of each variable to the predicted burst pressure.

## Results

### Performance according to device type and operator experience

#### Suture burst pressure

**Fig. 4:**
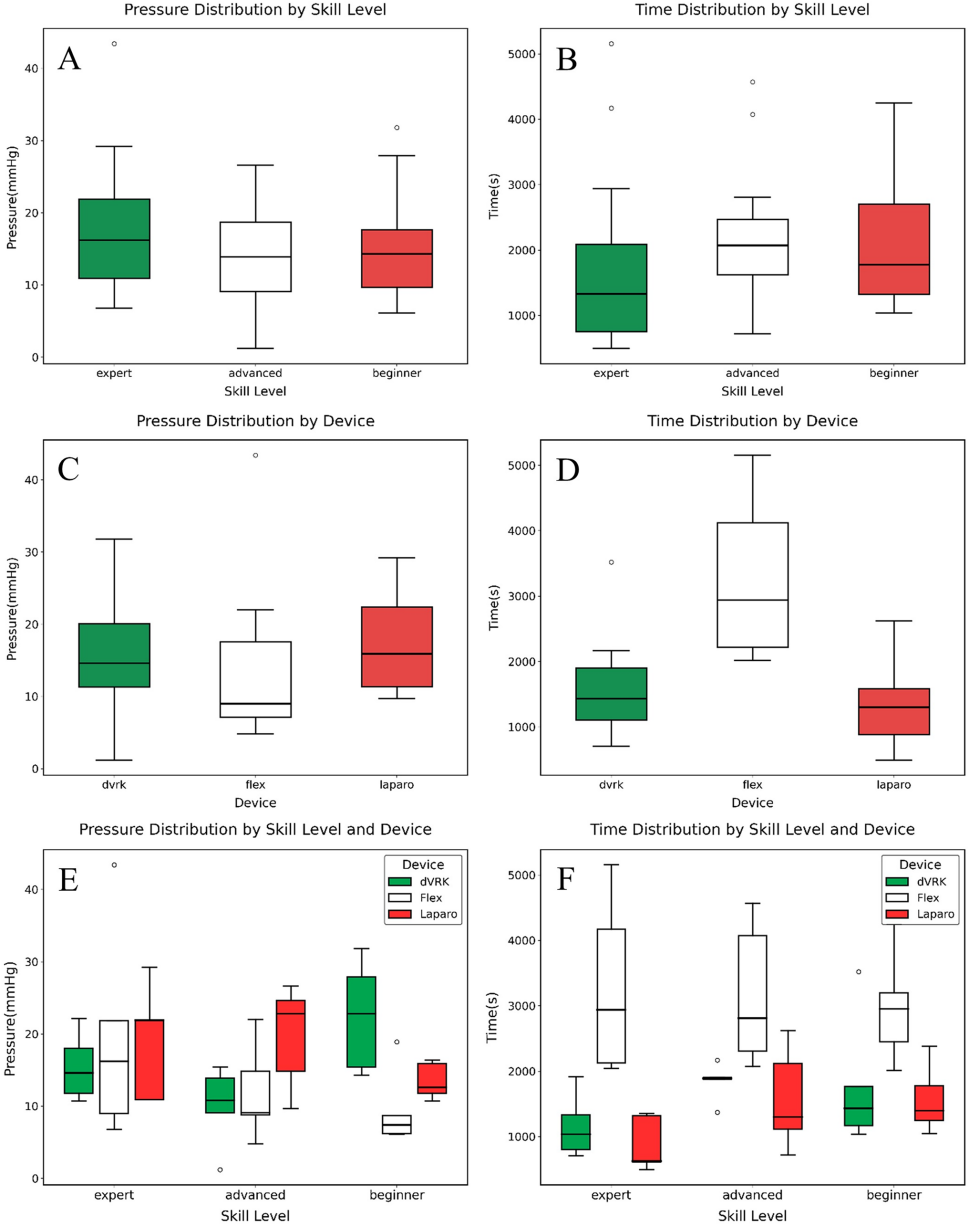
Differences in performance between device type and operator experience-level burst pressure distribution by device **A**; Burst pressure distribution by experience level **B**; Burst pressure distribution by device and experience level **C**; Time of task execution by device **D**; Time of task execution by experience level **E**; Time of task execution by device and experience level **F**. Outliers were calculated according to the IQR method and represented as dots

When comparing burst pressure across devices, no significant differences were found when outliers were included (p = 0.076). However, upon removal of one outlier using the interquartile range (IQR) method, a statistically significant difference emerged (p = 0.024), with post hoc testing indicating a significant superiority of laparoscopy compared to Flex (p = 0.029) and no further differences among groups (Table 2). Despite this finding, the sample size was insufficient to support robust conclusions. An estimated 81 to 223 participants per group would be required to achieve 80% statistical power, depending on the specific variable examined (Fig. 4A).

**Table 2:** Main Measurements by Device and Category

Device	Pressure^a^	Time^b^	PM^c^	PP	PM error	PP error
dVRK	15.99 ± 7.69	1594.53 ± 696.52	163.98 ± 76.13	179.74 ± 72.86	1.41 ± 1.05	1.44 ± 1.30
Flex	13.60 ± 10.08	3141.13 ± 1043.83	196.21 ± 114.29	214.58 ± 108.90	2.19 ± 1.83	2.08 ± 1.97
Laparo	17.38 ± 6.54	1341.13 ± 643.89	205.49 ± 103.96	207.73 ± 84.95	1.84 ± 1.52	1.55 ± 1.27
Category	Pressure	Time	PM	PP	PM error	PP error
Beginner	15.13 ± 7.64	2108.20 ± 991.83	193.02 ± 93.97	214.07 ± 78.50	1.82 ± 1.42	1.51 ± 1.33
Advanced	13.89 ± 7.41	2194.07 ± 1029.90	173.18 ± 115.07	169.75 ± 95.12	1.93 ± 1.84	1.89 ± 1.73
Master	17.95 ± 9.43	1774.53 ± 1368.94	199.48 ± 90.38	218.24 ± 91.71	1.69 ± 1.29	1.67 ± 1.61

^a^Pressure measurements are expressed in millimeters of mercury (mmHg);

^b^Time in seconds (s);

^c^PM, PP and their respective errors in pixels (px)

Stratification by operator experience (Beginner, Advanced, Master) revealed no significant differences in pressure performance, both with (p = 0.54) and without (p = 0.65) two outliers (Table 2). These findings were further corroborated by power analysis, which indicated low sensitivity for detecting potential group differences under the current sample conditions, particularly between 70 and over 500 sutures to achieve significance (Fig. 4B).

Analysis of the interaction between device type and operator category yielded one significant finding: among beginner participants, the dVRK was associated with significantly higher burst pressures compared to Flex (p = 0.03) (Fig. 4C).

### Task duration

Statistically significant differences in execution time were identified across devices (p < 0.01) before and after one outlier removal. Post hoc comparisons demonstrated that Flex was associated with significantly longer task times compared to both laparoscopy and dVRK. No significant difference was observed between dVRK and laparoscopy. The required sample size to detect it was estimated at 111 participants, highlighting the limited statistical power for this comparison (Fig. 4D).

Regarding operator experience, no significant differences were observed prior to outlier exclusion. After removing four outliers, the Kruskal–Wallis test revealed significant differences (p < 0.05) between experts and both beginners and advanced users (Fig. 4E).

When stratifying by both platform and experience level, statistically significant differences in task duration were identified between Flex and laparoscopy in both the advanced and expert subgroups, but not in the beginner subgroup (Fig. 4F).

### Subjective usability and workload assessment

System Usability Scale (SUS) scores were evaluated using either one-way ANOVA or the Kruskal–Wallis test. A significant difference in perceived usability was observed across platforms (p = 0.003). Post hoc analysis indicated that Flex received significantly lower usability ratings compared to both da Vinci Research Kit (dVRK) and conventional laparoscopy, while no significant difference emerged between the latter two platforms. The operator experience level did not significantly affect SUS scores (p = 0.069).

The NASA Task Load Index (NASA-TLX) revealed statistically significant platform-related differences in several areas (p < 0.05), indicating that perceived cognitive and physical demands varied between devices. Higher levels of frustration and mental demand were observed when using the Flex system compared to the other platforms, while comparisons involving laparoscopy and dVRK yielded no significant differences.

### Quantitative relationship between suture accuracy and pressure resistance

#### Correlation analysis

Spearman’s correlation analysis identified eight out of eighteen suture-spacing metrics exhibiting statistically significant negative associations with burst pressure (Table 3).

**Table 3:** Distance metrics associated with burst pressure strength

Metric	Definition	ρ (Spearman)	*p* value
PP std	Variability in vertical distance	−0.375	0.011
PM mean error	Mean deviation from ideal horizontal spacing	−0.353	0.017
P all std	Overall variability in distances	−0.330	0.027
PM std	Variability in horizontal distance	−0.329	0.027
P all max error	Maximum deviation from all ideal spacing	−0.311	0.038
PM std error	Variability in deviation from horizontal ideal spacing	−0.308	0.039
P all mean error	Mean deviation from all ideal spacing	−0.307	0.040
P all std error	Variability in deviation from all ideal spacing	−0.295	0.049
Time	Seconds between first and last action	−0.295	0.049

Out of the eighteen metrics evaluated, eight were found significantly correlated with suture resistance (p < 0.05) and selected for further statistical analysis using regression

Despite significance (p < 0.05), post hoc power analysis revealed the study was underpowered. Achieving 80% power would require 51–85 sutures for the eight variables with p < 0.05 and up to over 2000 for the remaining ten non-significant metrics. While these correlations suggest a link between suture precision and mechanical resistance, inferential limitations persist. Regression modeling was therefore employed to assess predictive utility beyond associative trends.

#### Regression analysis

Substantial variability was revealed in predictive performance, with only 10 out of 50 folds yielding a positive coefficient of determination (R^2^ > 0.1), while the overall model performance was poor (R^2^ = -0.42 and MSE = 93.7), suggesting that it fails to explain variance in the target. This instability likely arises from data noise, given the wide range of MSE values (26.8–232.3) across folds. Residual analysis revealed systematic errors, particularly for extremes: low actual values (e.g., 1.2) were overpredicted (residual = -16.9), while high values (e.g., 43.4) were underpredicted (residual = 25.5). Finally, SHAP analysis identified “P all max error” (i.e., the overall maximum deviation from ideal spacing) and “PM mean error” (i.e., the mean deviation from ideal horizontal spacing) as the most influential features (mean |SHAP|: 1.86 and 1.85). However, their pronounced multicollinearity (VIF > 10) may lead to inflated coefficient variance.

## Discussion

It is essential to contextualize the findings presented in the “Differences According to Device Type and Operator Experience” section in light of a particular aspect of the study design. While participants were divided based on prior experience in minimally invasive surgery, this categorization predominantly reflected laparoscopic practice, as it remains the standard approach at our institution. Consequently, an asymmetry in familiarity with the surgical platforms involved in this study was introduced: variable with laparoscopy, limited with the daVinci Research Kit, and null with the Flex system. So, although experts had substantially greater training in laparoscopy, group experience levels were more comparable in dVRK and equivalent in Flex. This distribution enabled not only the assessment of the transferability of surgical skills from laparoscopic to robotic-assisted modalities, but also the ease of approach and intuitiveness of novel platforms stratified per operator background. As a result, these factors could, at least partially, explain the observed inferiority of Flex compared to the other platforms.

Additionally, participants may have been subject to a cognitive bias of “involvement” in the study. Therefore, both beginner and advanced operators put considerable effort into the proficient completion of the task, whereas more confident experts aimed for a satisfactory, ordinary result. This could partially explain the lack of difference in internal pressure resistance between experience levels.

Among beginners, the higher dVRK burst pressures compared to Flex may be interpreted as a strong argument for the greater ease of use at first approach of daVinci compared to other robotic platforms such as Flex. Notably, the significant differences in task duration between Flex and laparoscopy in both the advanced and expert subgroups, but not in beginners, may reflect that both platforms were equally unfamiliar to novice users, thereby minimizing relative performance disparities.

The regression analysis reveals fundamental limitations likely stemming from two key issues. First, severe multicollinearity among predictors (VIF > 10) inflates coefficient variance and obscures genuine relationships between features. Second, residual analysis indicates that the model fails to predict particularly extreme values. While the modest regression coefficient may reflect a limited sample size, the observed multicollinearity might also suggest further model misspecification arising from unaccounted nonlinear relationships, confounding variables, or, possibly, irreducible noise in the pressure measurement process.

Beyond its immediate scientific relevance, this work holds unprecedented translational potential to bridge critical gaps in surgical practice. While several other studies have demonstrated the feasibility of computer vision-based suture quality evaluation, none have established the quantitative relationship between image-derived spatial and geometric suture characteristics (e.g., stitch spacing and derivate metrics) and mechanical resistance properties, nor have they systematically compared performance across different experience levels and minimally invasive surgical platforms. If a robust quantitative relationship between stitch placement and pressure resistance can be established, this would not only deepen our understanding of what constitutes a mechanically sound suture but would also pave the way to developing intelligent surgical support systems. These could provide on screen, intraoperative feedback on suture quality, augmenting the surgeon’s perception and potentially improving outcomes, particularly in high-risk settings. Our vision envisions a future where data-driven metrics enhance surgical training and execution, bringing objectivity and precision to domains that have historically relied on experience alone.

### Limitations

First, computer vision-based spatial metrics provide objective measures of suture geometry but do not capture subsurface phenomena such as needle trajectory through tissue, tissue trauma, or mechanical forces during suturing: variables that may influence integrity. Second, burst pressure testing is subject to “weakest link” mechanics, whereby failure typically initiates at the most vulnerable point rather than reflecting aggregate suture quality. While this property may attenuate correlations between mean spatial metrics and burst pressure, it paradoxically enhances the translational relevance of our findings. Anastomotic leaks in practice result from focal mechanical failure at vulnerable sites rather than uniform construct degradation, making burst pressure a more realistic predictor of surgical outcome than measures reflecting average suture quality alone.

Considerably, power analyses revealed that the study was frequently underpowered (power < 0.80) to detect subgroup differences, particularly across experience levels and platform types. As such, while specific trends are promising, conclusions must be drawn with caution. Although geometric regularity may play a role in suture quality, a more comprehensive assessment incorporating additional biomechanical and contextual variables is warranted. Larger, well-powered studies will be essential to define clinically useful predictors of suture strength and to guide the development of intelligent intraoperative feedback systems.

## Conclusions

This study assessed suturing performance across three minimally invasive surgical platforms and three experience groups, focusing on burst pressure, task duration, subjective workload, and spatial accuracy. In this preliminary evaluation, significant differences in burst pressure emerged only between laparoscopy and Flex, with laparoscopy demonstrating superior mechanical resistance. Novice operators achieved higher burst pressures on dVRK compared to Flex, suggesting a potentially smoother initial learning curve with rigid robotics compared to flexible systems, though further investigation is needed to confirm this pattern. Within our study sample, experience was not associated with variations in suture resistance. Task execution time was significantly longer on Flex, and expert surgeons consistently completed procedures more rapidly across all systems. Subjective workload assessments indicated greater mental demand and frustration associated with Flex across all experience levels.

Among spatial metrics, greater irregularity in suture spacing correlated significantly with reduced pressure resistance (ρ < 0, p < 0.05), supporting its relevance as a potential indicator of suture quality. However, the regression analysis demonstrated limited predictive accuracy (R^2^ < 0.1), indicating that spatial parameters alone may not be sufficient for reliably predicting suture integrity, and highlighting the need for further exploration of complementary metrics.

## Supplementary Information

Below is the link to the electronic supplementary material.

Supplementary file1 (DOCX 22 KB)
