# Supplementary material for: From precision to strength: computer vision for suture quality assessment—an ex vivo pilot study
Source: Surg Endosc. 2025 Dec 4;40(3):1913–24. doi: 10.1007/s00464-025-12441-6 (PMC12971847; doi:10.1007/s00464-025-12441-6)
Supplement: Supplementary file 1 — Supplementary file1 (DOCX 22 KB) [file 464_2025_12441_MOESM1_ESM.docx]

TABLE 2. Cross Correlation Analysis

| A | **Time** | **PM mean** | **PM std** | **PP mean** | **PP std** | **PM max** | **PP max** | **P all mean** | **P all std** | **P all max** |
| --- | --- | --- | --- | --- | --- | --- | --- | --- | --- | --- |
| **Time(s)** | 1 | 0.087 | 0.48 | 0.274 | 0.519 | 0.352 | 0.43 | 0.186 | 0.454 | 0.409 |
| **PM mean** | 0.087 | 1 | 0.567 | 0.636 | 0.446 | 0.847 | 0.524 | 0.922 | 0.635 | 0.758 |
| **PM std** | 0.48 | 0.567 | 1 | 0.785 | 0.765 | 0.865 | 0.797 | 0.735 | 0.922 | 0.843 |
| **PP mean** | 0.274 | 0.636 | 0.785 | 1 | 0.787 | 0.779 | 0.919 | 0.885 | 0.813 | 0.885 |
| **PP std** | 0.519 | 0.446 | 0.765 | 0.787 | 1 | 0.657 | 0.901 | 0.663 | 0.854 | 0.809 |
| **PM max** | 0.352 | 0.847 | 0.865 | 0.779 | 0.657 | 1 | 0.713 | 0.901 | 0.846 | 0.919 |
| **PP max** | 0.43 | 0.524 | 0.797 | 0.919 | 0.901 | 0.713 | 1 | 0.776 | 0.851 | 0.887 |
| **P all mean** | 0.186 | 0.922 | 0.735 | 0.885 | 0.663 | 0.901 | 0.776 | 1 | 0.79 | 0.899 |
| **P all std** | 0.454 | 0.635 | 0.922 | 0.813 | 0.854 | 0.846 | 0.851 | 0.79 | 1 | 0.903 |
| **P all max** | 0.409 | 0.758 | 0.843 | 0.885 | 0.809 | 0.919 | 0.887 | 0.899 | 0.903 | 1 |
| **PM mean error** | 0.257 | 0.243 | 0.514 | 0.143 | 0.322 | 0.405 | 0.22 | 0.216 | 0.556 | 0.354 |
| **PP mean error** | 0.431 | -0.081 | 0.154 | 0.097 | 0.461 | 0.074 | 0.257 | -0.004 | 0.287 | 0.199 |
| **PM std error** | 0.417 | 0.18 | 0.469 | 0.167 | 0.383 | 0.389 | 0.252 | 0.191 | 0.47 | 0.376 |
| **PP std error** | 0.553 | -0.08 | 0.289 | 0.22 | 0.559 | 0.125 | 0.441 | 0.059 | 0.397 | 0.298 |
| **PM max error** | 0.304 | 0.268 | 0.526 | 0.129 | 0.296 | 0.453 | 0.211 | 0.225 | 0.507 | 0.367 |
| **PP max error** | 0.443 | -0.082 | 0.202 | 0.137 | 0.488 | 0.098 | 0.325 | 0.016 | 0.322 | 0.238 |
| **P all mean error** | 0.423 | 0.093 | 0.402 | 0.145 | 0.48 | 0.288 | 0.292 | 0.126 | 0.511 | 0.335 |
| **P all std error** | 0.543 | 0.048 | 0.416 | 0.217 | 0.529 | 0.278 | 0.39 | 0.135 | 0.48 | 0.372 |
| **P all max error** | 0.461 | 0.101 | 0.432 | 0.162 | 0.485 | 0.323 | 0.331 | 0.139 | 0.499 | 0.364 |
|  |  |  |  |  |  |  |  |  |  |  |

| B | **PM mean error** | **PP mean error** | **PM std error** | **PP std error** | **PM max error** | **PP max error** | **P all mean error** | **P all std error** | **P all max error** |
| --- | --- | --- | --- | --- | --- | --- | --- | --- | --- |
| **Time(s)** | 0.257 | 0.431 | 0.417 | 0.553 | 0.304 | 0.443 | 0.423 | 0.543 | 0.461 |
| **PM mean** | 0.243 | -0.081 | 0.18 | -0.08 | 0.268 | -0.082 | 0.093 | 0.048 | 0.101 |
| **PM std** | 0.514 | 0.154 | 0.469 | 0.289 | 0.526 | 0.202 | 0.402 | 0.416 | 0.432 |
| **PP mean** | 0.143 | 0.097 | 0.167 | 0.22 | 0.129 | 0.137 | 0.145 | 0.217 | 0.162 |
| **PP std** | 0.322 | 0.461 | 0.383 | 0.559 | 0.296 | 0.488 | 0.48 | 0.529 | 0.485 |
| **PM max** | 0.405 | 0.074 | 0.389 | 0.125 | 0.453 | 0.098 | 0.288 | 0.278 | 0.323 |
| **PP max** | 0.22 | 0.257 | 0.252 | 0.441 | 0.211 | 0.325 | 0.292 | 0.39 | 0.331 |
| **P all mean** | 0.216 | -0.004 | 0.191 | 0.059 | 0.225 | 0.016 | 0.126 | 0.135 | 0.139 |
| **P all std** | 0.556 | 0.287 | 0.47 | 0.397 | 0.507 | 0.322 | 0.511 | 0.48 | 0.499 |
| **P all max** | 0.354 | 0.199 | 0.376 | 0.298 | 0.367 | 0.238 | 0.335 | 0.372 | 0.364 |
| **PM mean error** | 1 | 0.341 | 0.792 | 0.393 | 0.956 | 0.333 | 0.808 | 0.647 | 0.764 |
| **PP mean error** | 0.341 | 1 | 0.592 | 0.888 | 0.342 | 0.974 | 0.829 | 0.831 | 0.825 |
| **PM std error** | 0.792 | 0.592 | 1 | 0.619 | 0.806 | 0.574 | 0.842 | 0.888 | 0.833 |
| **PP std error** | 0.393 | 0.888 | 0.619 | 1 | 0.388 | 0.908 | 0.79 | 0.91 | 0.809 |
| **PM max error** | 0.956 | 0.342 | 0.806 | 0.388 | 1 | 0.343 | 0.783 | 0.651 | 0.795 |
| **PP max error** | 0.333 | 0.974 | 0.574 | 0.908 | 0.343 | 1 | 0.808 | 0.833 | 0.842 |
| **P all mean error** | 0.808 | 0.829 | 0.842 | 0.79 | 0.783 | 0.808 | 1 | 0.905 | 0.971 |
| **P all std error** | 0.647 | 0.831 | 0.888 | 0.91 | 0.651 | 0.833 | 0.905 | 1 | 0.911 |
| **P all max error** | 0.764 | 0.825 | 0.833 | 0.809 | 0.795 | 0.842 | 0.971 | 0.911 | 1 |
